# Identification of Neonatal White Matter on DTI: Influence of More Inclusive Thresholds for Atlas Segmentation

**DOI:** 10.1371/journal.pone.0115426

**Published:** 2014-12-15

**Authors:** Rachel L. Vassar, Naama Barnea-Goraly, Jessica Rose

**Affiliations:** 1 Department of Orthopaedic Surgery, Stanford University School of Medicine, Stanford, CA, United States of America; 2 Neonatal Neuroimaging Laboratory, Stanford University School of Medicine, Stanford, CA, United States of America; 3 Center for Interdisciplinary Brain Sciences Research, Stanford University School of Medicine, Stanford, CA, United States of America; Beijing Normal University, China

## Abstract

**Purpose:**

Semi-automated diffusion tensor imaging (DTI) analysis of white matter (WM) microstructure offers a clinically feasible technique to assess neonatal brain development and provide early prognosis, but is limited by variable methods and insufficient evidence regarding optimal parameters. The purpose of this research was to investigate the influence of threshold values on semi-automated, atlas-based brain segmentation in very-low-birth-weight (VLBW) preterm infants at near-term age.

**Materials and Methods:**

DTI scans were analyzed from 45 VLBW preterm neonates at near-term-age with no brain abnormalities evident on MRI. Brain regions were selected with a neonatal brain atlas and threshold values: trace <0.006 mm^2^/s, fractional anisotropy (FA)>0.15, FA>0.20, and FA>0.25. Relative regional volumes, FA, axial diffusivity (AD), and radial diffusivity (RD) were compared for twelve WM regions.

**Results:**

Near-term brain regions demonstrated differential effects from segmentation with the three FA thresholds. Regional DTI values and volumes selected in the PLIC, CereP, and RLC varied the least with the application of different FA thresholds. Overall, application of higher FA thresholds significantly reduced brain region volume selected, increased variability, and resulted in higher FA and lower RD values. The lower threshold FA>0.15 selected 78±21% of original volumes segmented by the atlas, compared to 38±12% using threshold FA>0.25.

**Conclusion:**

Results indicate substantial and differential effects of atlas-based DTI threshold parameters on regional volume and diffusion scalars. A lower, more inclusive FA threshold than typically applied for adults is suggested for consistent analysis of WM regions in neonates.

## Introduction

Diffusion tensor imaging (DTI) is a technique used to characterize water diffusion in the brain and investigate white matter (WM) microstructure and tissue orientation. Widely used in adult populations, DTI is now implemented in neonates for quantitative analysis of early WM development. Neonatal WM structure differs from WM in children and adults, and the optimal fractional anisotropy (FA) threshold used for WM segmentation may be different from the threshold of FA>0.25 commonly used in adults [1],[2]. WM tracts in neonatal brains are only partially developed and myelinated [3], and little is known about appropriate FA threshold values needed to discriminate these tracts. This study addresses this challenge and investigates the effect of segmentation parameters on atlas-based DTI analysis of developing WM in very-low-birth-weight (VLBW) preterm infants at near-term age.

Understanding typical developmental trajectories of WM in the neonatal brain is critical for identification of abnormalities that may indicate risk for future neurological impairments and functional deficits [4]. In preterm infants at near-term age, DTI values within the corticospinal tract and corpus callosum (CC) have demonstrated associations with later motor and cognitive function [5],[6],[7]. In addition, DTI may be more sensitive to WM injury in the neonatal brain than conventional MRI for diagnosis of cerebral palsy and prognosis of future function [8],[9]. VLBW preterm infants typically receive standard-of-care MRI brain scans prior to discharge from the neonatal intensive care unit (NICU). This may offer a clinically feasible opportunity to provide early prognosis that may guide interventions aimed at reducing neurodevelopmental problems in preterm children.

A semi-automated approach to region-of-interest (ROI) selection, rather than manual analyses, is important for clinical implementation of DTI analysis. Neonatal brain atlases have recently been developed to facilitate a more comprehensive, time-efficient approach to DTI analysis. Atlases, such as the one developed by Oishi et al. [10], based on a population of healthy neonates, provide semi-automated brain parcellation for MRI and DTI. Meaningful comparison of DTI measures across studies and NICUs will require replicable DTI techniques and consistent reporting, as different imaging protocols and analysis techniques may substantially influence results [2],[11]. The whole-brain atlases continues to be improved and allows for increased scan-rescan reproducibility and more accurate registration accuracy between each subject and the atlas, essential elements for clinical implementation of DTI [12],[13].

Diffusion imaging can be used to derive scalars calculated per voxel within the brain image. Commonly used scalars include FA, axial diffusivity (AD), and radial diffusivity (RD) values, and together these values allow for characterization of WM structure and development. Developing WM is expected to have lower FA values than adult WM [14],[15] and thus a lower FA threshold may be necessary for segmentation of neonatal WM.

Previous neonatal DTI studies have used a variety of segmentation techniques and threshold parameters for identification of regions and WM tracts. FA values ranging from FA>0.05 to FA>0.25 [14],[16],[17] have been used to delineate WM in the developing brain. Application of a higher FA threshold may restrict measurements primarily to myelinated, highly ordered regions of WM, whereas a lower threshold may allow for the inclusion of less mature WM regions that may better represent the progression of development and injury during the neonatal period. Too low of a threshold, however, may include grey matter (GM) and introduce partial volume effects. Distinguishing WM from GM is particularly challenging in the developing neonatal brain and this may introduce additional variability in WM segmentation. To date, threshold values for atlas-based brain segmentation in neonates have been based on adult values, and the effect of different thresholds on neonatal diffusion measurements has not yet been quantified. This study aims to examine how the application of different FA thresholds may alter regional volume selected and diffusion measurements obtained from atlas-based brain segmentation in neonates.

## Materials and Methods

### Subjects

Participants (n = 45) included in this study had no brain abnormalities evident on near-term clinical evaluation of structural MRI and represent a sub-population of 102 VLBW preterm infant participants who received standard-of-care MRI prior to discharge from the Lucile Packard Children’s Hospital NICU. IRB approval was obtained for this study from the Administrative Panel on Human Subjects in Medical Research at the Stanford University Research Compliance Office. Parents of infant participants were approached prior to scheduled MRI, and written, informed consent was obtained for participation in this study of neurodevelopment. The IRB approved the methodology of consent from the parents of the infants, as the infant participants were too young to legally consent to participation in research. Protocol ID No: 13899 IRB Approval Number: 6208.

Inclusion criteria were: gestational age (GA) at birth ≤32 wks, birth-weight ≤1500 g, and no evidence of genetic disorders or congenital brain abnormalities. 66 of the total 102 participants had successful DTI scans collected at the end of standard-of-care MRI, before discharge from the NICU. This analysis includes a subset of infants (n = 45) who received MRI scans at ≤40 wks PMA and had no evidence of brain abnormalities on MRI as reported by the attending clinical neurologist and confirmed by neuroradiologist X.S. The demographic characteristics of all participants with DTI (n = 68) and the sub-population with DTI and normal MRI findings (n = 45) are shown in **Table 1**.

**Table 1:** Population demographics.

	All neonates with DTI (n = 66)	Neonates with DTI, no brain abnormalities on MRI, scan <40 wks PMA (n = 45)
Males/Females, n (%)	25/41 (38%/62%)	17/28 (38%/62%)
Birthweight (g), mean (SD)	1090 (266)	1092 (261)
GA-at-birth (wks), mean (SD)	28.9 (2.3)	28.9 (2.3)
PMA-at-scan (wks), mean (SD)	36.5 (1.3)	36.3 (1.0)

### MRI data acquisition

Brain MRI scans were performed on 3T MRI (GE- Discovery MR750, GE 8-Channel HD head coil) at Lucile Packard Children’s Hospital at Stanford University. The MRI included T1-weighted, T2-weighted, diffusion-weighted imaging (DWI) and diffusion tensor imaging (DTI) scans. A gradient echo 3-plane localizer was used, and an asset calibration was proscribed to utilize parallel imaging. From the 3-plane localizer, sagittal T1 FLAIR images were generated with the following parameters: TE = 9l.0, TR = 2200, FOV = 20 cm, matrix size = 320×224, slice thickness 3.0×0.5 mm spacing, NEX = 1. T2, DWI, and DTI axial scans were proscribed using a single acquisition, full phase field of view (FOV). The Fast Recovery, Fast Spin Echo T2 scan imaging parameters were: TE = 85 ms, TR = 2500, FOV 20 cm, matrix = 384×224; slice = 4.0 mm×0.0 mm spacing. Axial T2 FLAIR parameters were: TE = 140, TR = 9500, FOV = 20 cm, slice = 4.0 mm×0.0 mm, inversion time 2300 fluid attenuated inversion recovery matrix = 384×224. Axial DWI parameters were: TE = 88.8, TR = 10,000, FOV = 20 cm, slice = 4.0 mmx0.0 spacing, matrix = 128×128. The DTI scan was based on a diffusion-weighted, single-shot, spin-echo, echo-planar imaging sequence with a slice thickness of 3.0 mm, a matrix size of 128×128, a 90° flip angle, FOV = 20 cm, TE = 88.8 ms, TR = 8000, (b = 1000 s/mm^2^). Diffusion was measured along 25 directions, with three B0 images. Two repetitions were obtained from 64/66 subjects. MRI scans were performed as routine near-term neuroimaging for preterm infants and the diffusion-weighted sequences were collected at the end of these ∼25-minute MRI scans. Infants were swaddled and fed and typically remained asleep for the duration of the scan. Sedation was not utilized as part of the research protocol and typically was not utilized for routine near-term MRI, although it may have been prescribed according to clinical needs in some cases.

### DTI processing

Diffusion-weighted images were pre-processed using DTI Studio [18]. The best repetition was selected to eliminate images with artifacts or evidence of motion for infants with two repetitions (n = 64/66). If no full repetition was usable, a composite repetition was generated based on best image slices. An infant’s DTI scan was included if at least one quality image was obtained for each slice from either repetition. Infants were excluded if neither repetition provided a viable image for any given slice or if it was not possible to combine the two repetitions due to head orientation differences between the two repetitions. Automated Image Registration was performed in DTI Studio using an affine transformation to further correct for eddy current distortions and motion.

Each subject’s DTI brain scan was skull-stripped with ROI Editor using the B0 and trace images and manually rotated to align with the vertical JHU neonatal template image. The DTI images were then processed with DiffeoMap (www.mristudio.org) using the FA and trace maps to perform a large deformation diffeomorphic metric mapping (LDDMM) transformation [10]. Each brain was normalized to map onto the neonatal atlas (http://cmrm.med.jhmi.edu/) and segmented into 126 regions based on the individual brain’s anatomical markers and diffusion patterns. On the trace map, regions with trace >0.006 mm^2 ^s^−1^ corresponding to CSF were excluded from the segmented regions originally defined by the atlas. Threshold values of FA>0.15, FA>0.20, and FA>0.25 were then applied to the FA, AD, and RD maps of each subject, and regional mean FA, AD, and RD values were obtained.

The FA thresholds used in this study were selected based on recommendations for neonatal and adult WM tracts. Developers of the JHU atlas suggest use of the threshold FA>0.25 to identify adult WM fiber tracts [19]. Oishi et al. also suggests that adult cortical GM can be reliably excluded with a FA>0.15 threshold, as cortical GM almost always has FA<0.15 [19]. Furthermore, past neonatal DTI studies using semi-automated segmentation techniques have primarily used FA thresholds in the range of FA>0.15 to FA>0.25.

**Figure 1:**
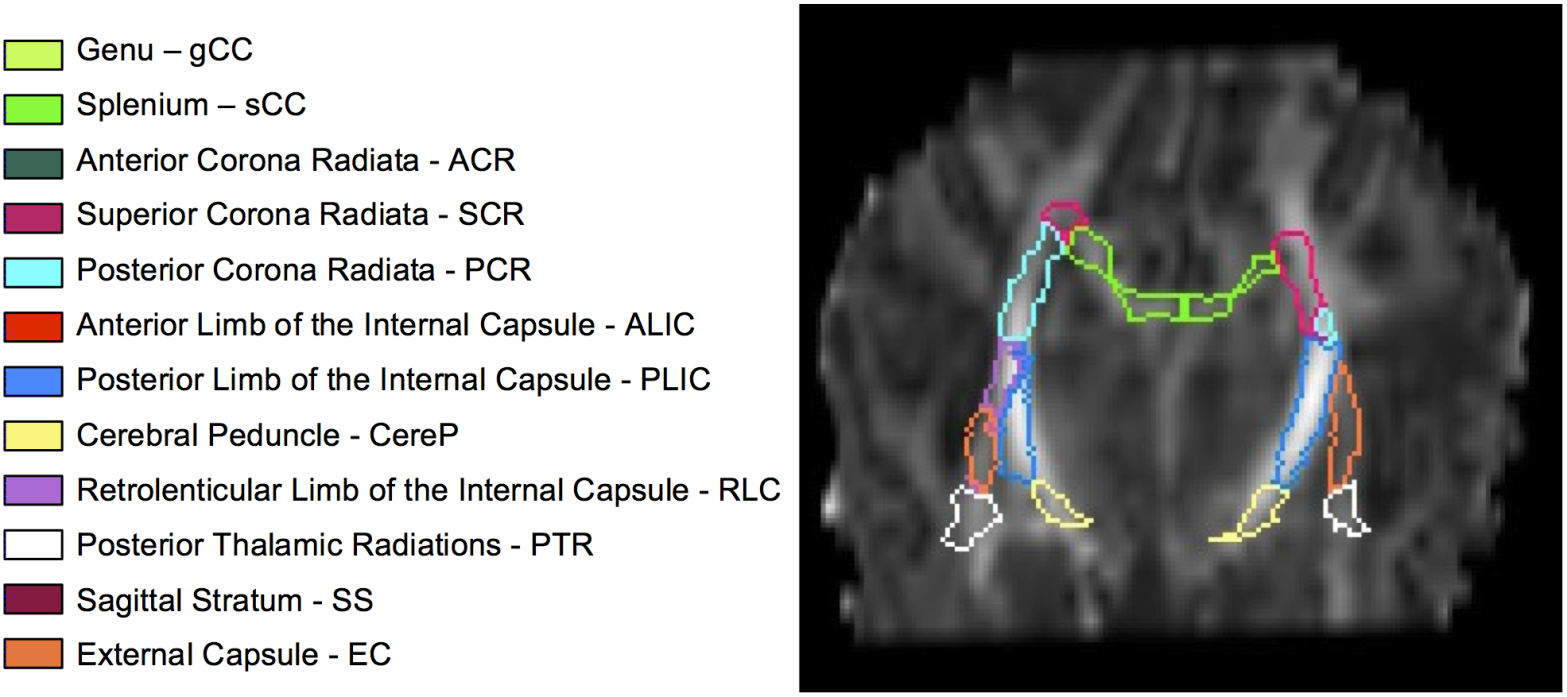
Twelve subcortical white matter regions and their corresponding color designations, as segmented in a representative infant.

**Figure 2:**
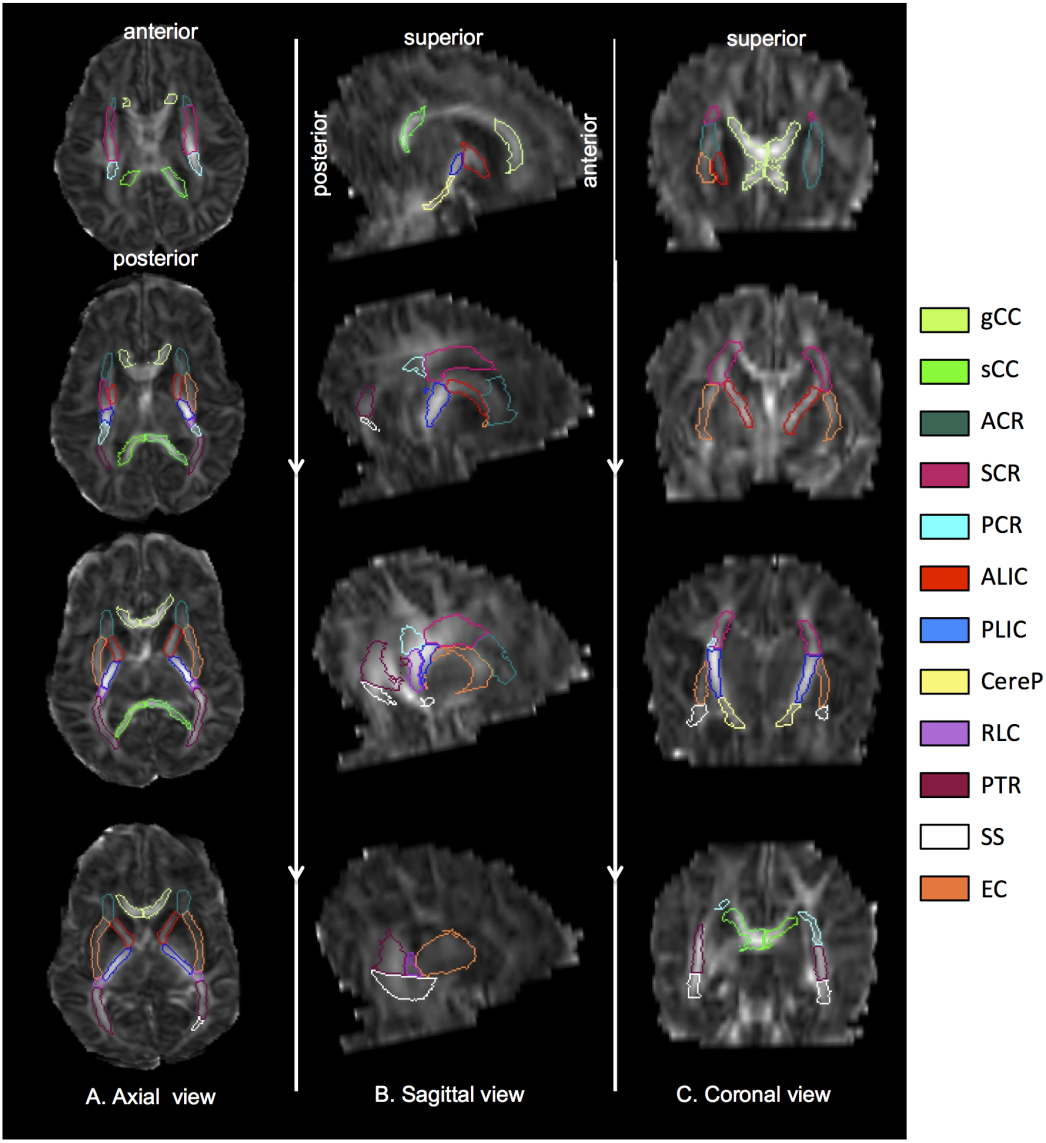
Brain images acquired from a representative scan, A: axial slices from superior to inferior, B: sagittal slices from medial to lateral, and C: coronal slices from anterior to posterior. ROIs were obtained from LDDMM DiffeoMap registration process and visualized onto the FA maps in ROIEditor. Regions were defined by the JHU neonatal atlas and the trace threshold.

Brain region volumes selected using three FA thresholds were reported as a percent of regional volumes obtained with the neonatal atlas after exclusion of CSF with the trace <0.006 threshold. Regions with fewer than 10 voxels selected were excluded. Regions analyzed included WM tracts thought to be undergoing development during the neonatal period and that are related to motor and cognitive function. Brain regions are listed in **Fig. 1**, along with images of their positions within the brain (**Fig. 2**) according to the JHU neonatal atlas.

### Statistical analysis

Regional brain volumes defined by the FA>0.15, FA>0.20, and FA>0.25 thresholds were calculated and reported as a mean percentage ±95% confidence intervals (CI) of the original regional volumes, as defined after exclusion of CSF with the trace <0.006 threshold. Coefficients of variability (CV) were calculated for each region as segmented with four thresholds. After defining a region by a given threshold, the FA, AD, and RD mean value and standard deviation (n = 45) were determined. CV for each region and for each threshold was then calculated as the ratio of the standard deviation/mean, which is a normalized measure of the distribution and variability of the data points.

FA, AD, and RD mean ±95% CI values were calculated for each of the twelve WM regions, bilaterally. Within each region, mean DTI values as defined by trace <0.006, FA>0.15, FA>0.20, and FA>0.25 were compared using a non-parametric Kruskal-Wallis one-way analysis of variance (ANOVA), performed in Excel with the XLSTAT extension. Significant differences were defined as p<0.00417, corrected for multiple comparison in 12 separate regions.

## Results

### Regional Volumes

**Figure 3:**
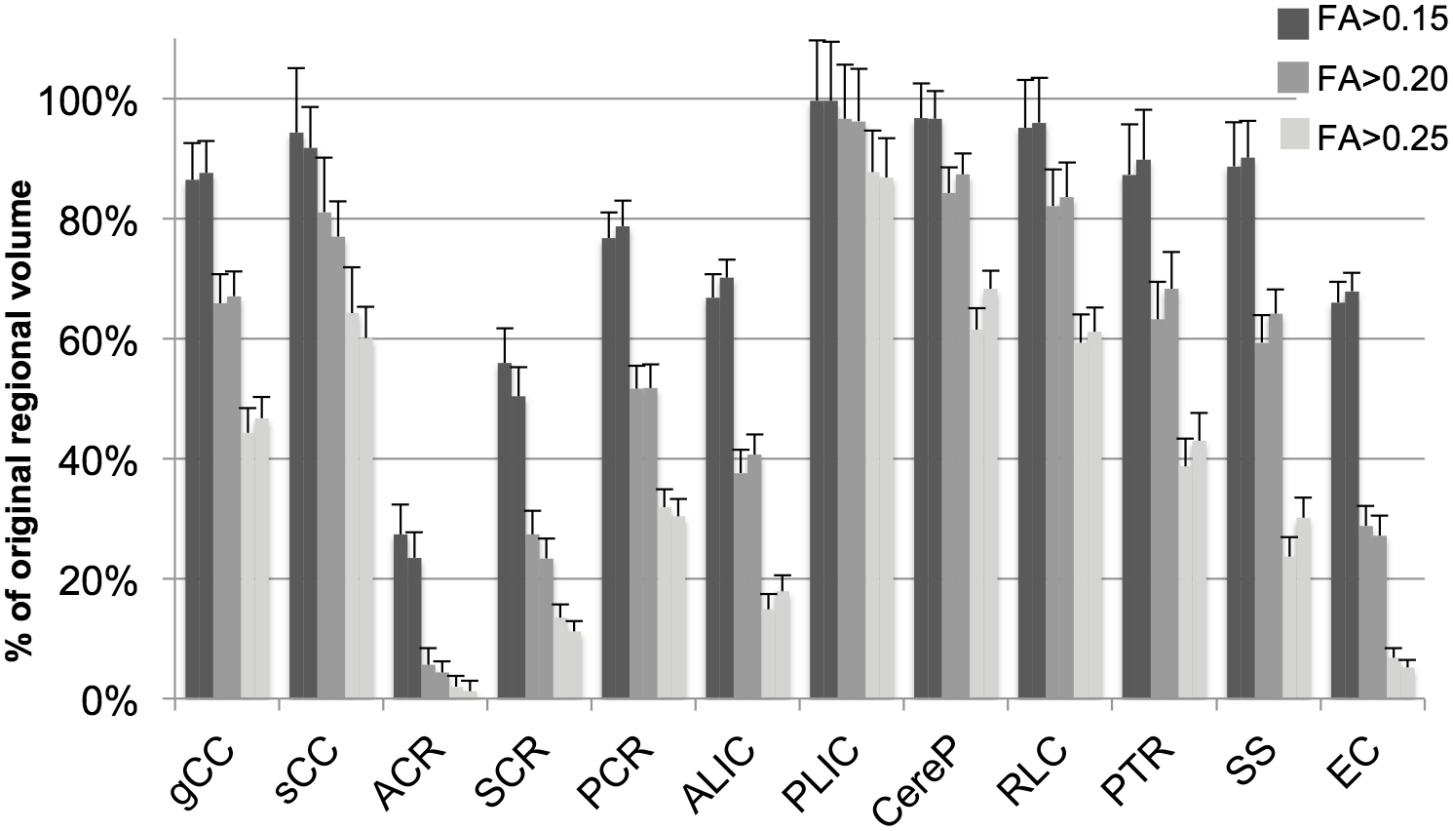
Volume of WM regions selected with three FA thresholds, reported as a percent of total volume as defined by the neonatal atlas and trace threshold (mean±95% confidence intervals), in the left and right hemispheres, represented by adjacent left and right bars.

The application of different thresholds resulted in significant differences in selected volumes in all brain regions assessed. **Fig. 3** shows the left and right regional volumes selected with threshold levels of FA>0.15, 0.20, and 0.25, reported as a percentage of total volume obtained with the neonatal atlas and trace threshold to exclude CSF. As expected, application of higher FA thresholds resulted in substantial reduction of brain region volumes selected for all regions, particularly for regions with mean FA values in the range of 0.15–0.25, such as the ALIC, corona radiata (CR) and EC. Regions with higher FA values, such as the PLIC, were least affected by the application of the three FA thresholds.

Application of the threshold FA>0.25 eliminated a substantial percentage of most regions. Compared to original regional volumes defined by the atlas, the threshold FA>0.25 resulted in a reduction in volume to a mean (range) of 37% (2.0–88%) on the left and 38% (1.1–87%) on the right. The application of the threshold FA>0.20 resulted in less substantial reductions in volume to 57% (5.6–97%) on the left; 58% (4.4–96%) on the right. The threshold FA>0.15 retained the largest regional volumes, averaging 78% (27–100%) on the left; 78% (23–100%) on the right and included more than 50% of all subcortical WM regions with the exception of the ACR.

### Variability of Selected Volumes

**Figure 4:**
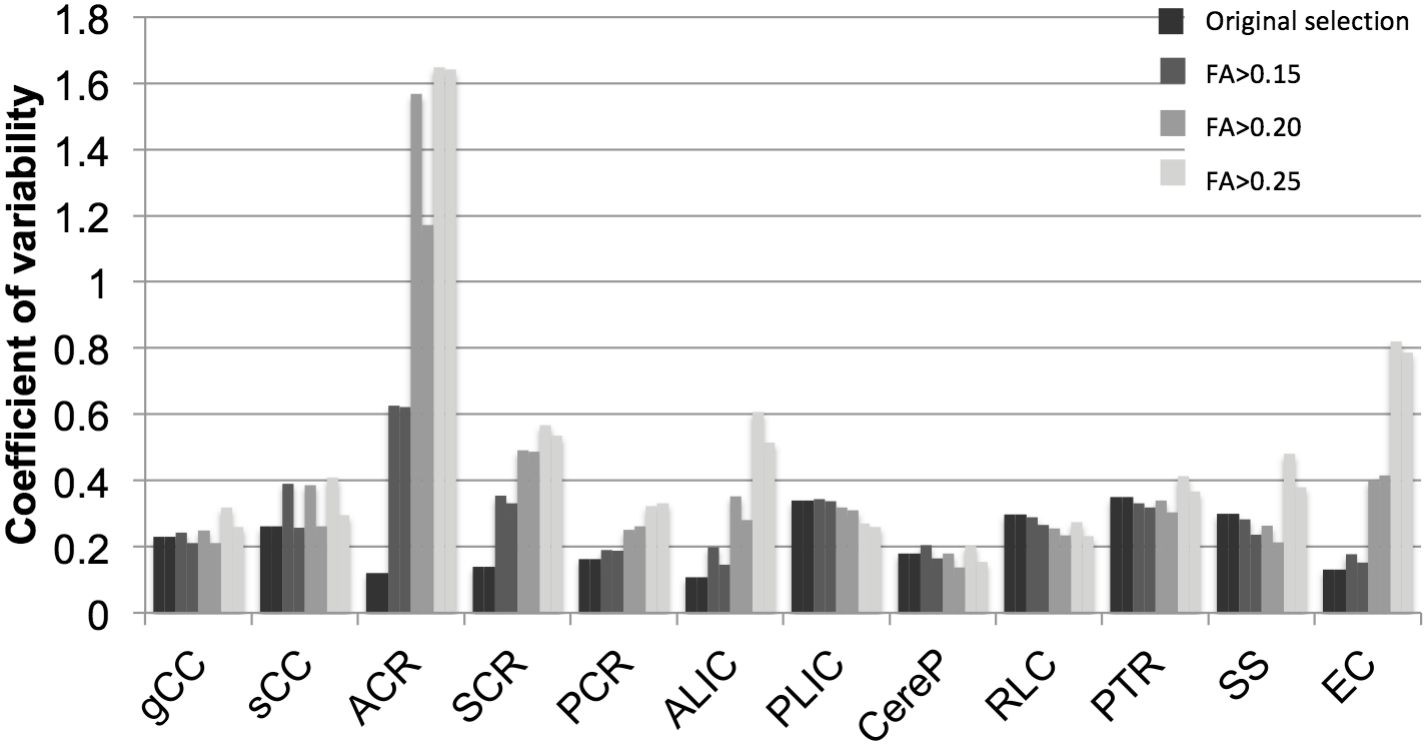
Coefficients of variability (CV) of regional WM volumes for total volume as defined originally by the neonatal atlas and trace threshold, and for volumes defined by FA>0.15, FA>0.20, and FA>0.25 thresholds in the left and right hemispheres represented by adjacent left and right bars.

In general, application of higher thresholds resulted in more inter-subject variability of selected regional volumes as evidenced by higher CV (**Fig. 4**). Mean CV was 0.24 with the trace threshold, 0.29 with threshold FA>0.15, 0.39 with threshold FA>0.20, and 0.50 with threshold FA>0.25. The PLIC, RLC, and CereP demonstrated the least change in CV with application of the different thresholds; the ACR, ALIC, and EC demonstrated the highest variability. Using the FA>0.25 threshold, the ACR could only be identified bilaterally in 1/45 subjects, compared to 45/45 with the FA>0.15 threshold.

### Regional FA, AD, and RD values

**Figure 5:**
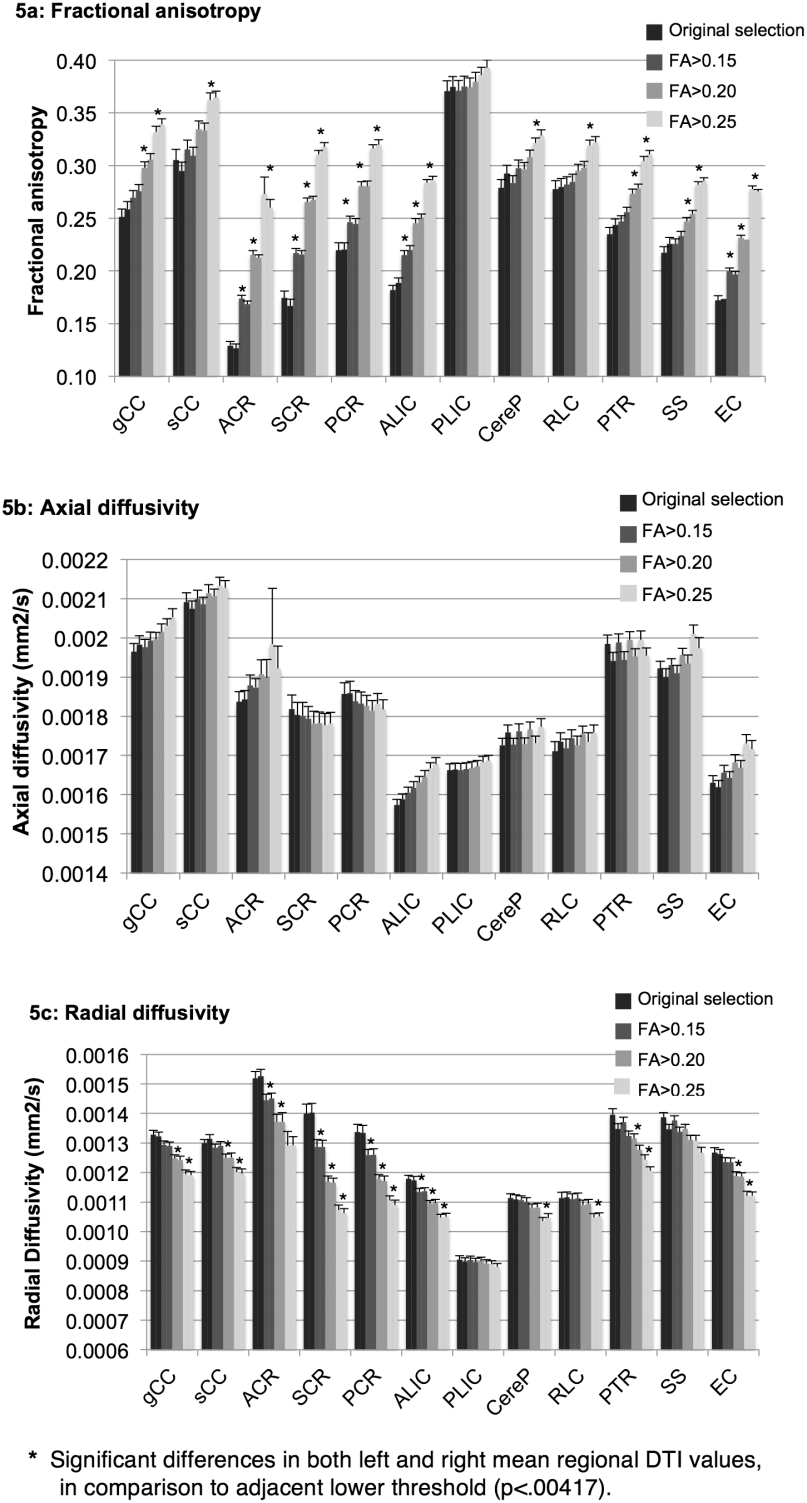
Regional WM mean DTI values measured using different FA thresholds. Mean (±95%CI) values of fractional anisotropy (a), axial diffusivity (b), and radial diffusivity (c), within WM regions originally selected with the neonatal atlas and trace <0.006 threshold, and with FA>0.15, FA>0.20, and FA>0.25 thresholds in left and right hemispheres represented by adjacent left and right bars.

**Fig. 5a–c** shows mean FA, AD, and RD values for twelve brain regions in the left and right hemispheres, obtained with the original atlas-defined regions after excluding CSF (trace <0.006 mm^2 ^s^−1^), as well as with the threshold levels of FA>0.15, 0.20, and 0.25. 95% confidence intervals are reported.

In general, application of the three progressively higher thresholds resulted in significantly higher mean FA values within each region, except for the in the PLIC, where FA values were not significantly different with application of the four thresholds (**Fig. 5a**). In the CereP and RLC, mean FA values were only significantly different when comparing the FA>0.20 and FA>0.25 thresholds. In the splenium, PTR, and SS, mean FA was significantly different when comparing the FA>0.15 vs. FA>0.20 threshold and the FA>0.20 vs. FA>0.25 threshold. The PLIC demonstrated the highest FA values with application of all thresholds, and the ACR demonstrated the lowest FA values. Furthermore, regions were unequally affected by application of higher thresholds: the PLIC, RLC, and CereP demonstrated the least change in FA values, as these regions generally had the highest FA. In contrast, FA values in the ACR, SCR, and EC were most affected by changes in applied thresholds, as these regions had relatively low FA values that were close to the threshold values.

Mean AD values demonstrated limited changes with application of the three thresholds (**Fig. 5b**). Values did not significantly differ with application of the various thresholds in the PLIC, CereP, RLC, PTR, CR, and splenium. For all applied thresholds, the CC demonstrated the highest AD values.

Mean RD values decreased significantly with the application of progressively higher FA thresholds in almost all WM regions (**Fig. 5c**) except for in the PLIC. In the CereP and RLC, RD values were only significantly different when comparing FA>0.20 vs. FA>0.25 thresholds. PLIC RD values were consistently lowest and were least affected by application of the three thresholds. The RD values of the ACR were consistently highest, and RD in the ACR, SCR, and PCR decreased most substantially with the application of higher threshold values.

## Discussion

In this study, application of higher FA thresholds resulted in more variable WM volume selection and significant changes in regional diffusion values of near-term WM. The FA threshold of FA>0.25 substantially reduced the amount of brain tissue selected (**Fig. 3**)**.** Brain regions were differentially affected by application of thresholds. In general, regions with low FA and high RD values such as the CR, ALIC, and EC were most affected by application of varying FA thresholds.

Regions with low FA and higher RD are likely the least developed WM regions in the neonatal brain [3]. Other explanations are also possible; for example, the adult CR is known to contain crossing fibers that results in artificially low FA measured on DTI, compared to the actual directionality of the multiple fiber tracts in that region [10],[15]. Regions with fibers at varying stages of development, or regions with crossing fibers, may be more challenging to segment and less useful for clinical analysis in the neonatal brain. Regions such as the CR, ALIC, and EC were observed in this study to have variable DTI measurements depending on segmentation parameters. DTI analysis in these regions during the neonatal period should be performed cautiously, as DTI values may be less informative about neuronal development and more manipulated by selected segmentation parameters. Conversely, the PLIC, CereP, and RLC demonstrated much less variation in DTI values with application of different thresholds, suggesting that these regions are more robust regions for neonatal DTI analysis, as they are further along in development and may be less affected by particular thresholds used for segmentation. Changes in DTI values in these regions may be more reflective of differences in WM integrity and directionality, and offer prognostic value in clinical settings.

The threshold FA>0.25 is commonly used to select WM in the adult brain, but this threshold may not be optimal for the neonatal brain, in which tracts are less organized and just beginning to myelinate [10],[20],[21]. The neonatal brain WM undergoes rapid change during the first postnatal months [15], thus, a degree of inter-subject variability in DTI measures is expected. Due to the high rate of change in DTI values during the near-term period [10],[15],[21],[24],[25],[26], segmentation thresholds should allow for inclusion of tracts at various stages of maturation. This study demonstrates that less inclusive, higher FA thresholds, such as FA>0.25 as typically used for adult WM segmentation, resulted in inconsistent selection of regions (**Fig. 4**), particularly for those regions with low FA and high RD such as the CR, ALIC, and EC. High threshold values exclude substantial portions of developing WM in the neonatal brain and may omit evidence of WM injury or regions with prognostic value. Lower, more inclusive thresholds than the standard of FA>0.25 used for adult WM segmentation may be more appropriate in the developing neonatal brain. Too low of a threshold, however, may increase the influence of partial volume effects due to inclusion of additional brain tissue or CSF adjacent to WM tracts [2],[22],[23]. Identifying an appropriate FA threshold to use for neonatal WM segmentation is important for repeatable and meaningful measurement of WM development.

Distinguishing WM tracts from surrounding tissues is particularly difficult in the neonatal brain. Compared to adults, neonatal brain analysis on DTI is even more challenging due to small brain size, tissue composition, and particularly limited acquisition time [2]. Due to the small brain size and low degree of myelination, partial volume effects and low signal-to-noise ratio (SNR) may impair the ability to accurately segment WM regions. These partial volume effects may influence tracts differently based on bundle thickness, orientation, and curvature [22]. Thus, identifying an optimal FA threshold for neonatal WM selection involves a balance between inclusion of developing WM while avoiding large influence from partial volume effects. Furthermore, the same FA threshold may not be appropriate for all WM tracts – more developed tracts such as those traveling through the PLIC, CereP, and RLC, may be better segmented with higher thresholds, or may be less dependent on threshold for segmentation. The FA>0.15 threshold may be better for less-well defined tracts in the neonatal brain. An atlas-based approach to regional analysis may minimize the influence of a low SNR and increases the statistical power of measurements.

A limitation of atlas-based segmentation is its reliance on an individual subject’s regional FA values to align the parcellation map; thus, gross brain abnormalities may influence segmentation [10]. For this study, participants had no brain abnormalities evident on clinical evaluation of structural MRI, in order to limit influence of the lower accuracy of atlas-based segmentation of brains with known anatomical deviations. Atlas alignment was also inspected manually on each brain in the analysis. Despite the difficulties of regional parcellation in the neonatal brain, the JHU neonatal atlas has demonstrated a high level of agreement with manual segmentation techniques [10]. Several neonatal brain atlases exist for regional segmentation, and this study was limited in its assessment of only one atlas. The JHU neonatal atlas, however, is a representative example of atlases used for DTI analysis that could be clinically implemented. Differences in scanners and protocols may also affect regional segmentation and the optimal parameters for neonatal WM analysis. Further studies are needed to determine optimal thresholds and repeatable methods for clinical implementation of DTI analysis of neonatal WM development.

In conclusion, the optimal threshold for neonatal WM segmentation differs from those recommended for use in adults due to differences in WM structure and myelination. Application of the neonatal atlas and three different FA thresholds used to select brain regions resulted in significant differences in percent regional volume selected and DTI values. The smaller, more variable volumes selected using standard adult FA threshold values suggest that lower, more inclusive FA thresholds should be used to investigate developing neonatal WM. Additionally this study identifies the extent to which various regions are affected by segmentation parameters and suggests WM tracts that may be more robust for repeatable analysis in the neonatal period. The challenges in neonatal brain imaging and analysis of near-term DTI underscore the importance of application of evidence-based methods that may increase feasibility and prognostic value of DTI in a clinical setting. This is one of the first studies to quantify the effect of different thresholds on atlas-based regional selection and DTI measurements of WM in the neonatal brain. Consistent, automated methods for DTI segmentation and analysis are important for the clinical implementation of near-term brain DTI that may detect subtle developmental abnormalities and risk for future developmental impairments.

## Supporting Information

S1 Table **Mean fractional anisotropy, axial diffusivity, and radial diffusivity values for twelve bilateral regions segmented using four thresholds, including trace <0.006, FA>0.15, FA>0.20, and FA>0.25.**
(XLSX)
